# Epigenetic inheritance of an inducibly nucleosome-depleted promoter and its associated transcriptional state in the apparent absence of transcriptional activators

**DOI:** 10.1186/1756-8935-2-11

**Published:** 2009-09-11

**Authors:** Ryosuke Ohsawa, Melissa Adkins, Jessica K Tyler

**Affiliations:** 1 Department of Biochemistry and Molecular Genetics, University of Colorado School of Medicine, Aurora, CO, USA

## Abstract

**Background:**

Dynamic changes to the chromatin structure play a critical role in transcriptional regulation. This is exemplified by the Spt6-mediated histone deposition on to histone-depleted promoters that results in displacement of the general transcriptional machinery during transcriptional repression.

**Results:**

Using the yeast *PHO5 *promoter as a model, we have previously shown that blocking Spt6-mediated histone deposition on to the promoter leads to persistent transcription in the apparent absence of transcriptional activators *in vivo*. We now show that the nucleosome-depleted *PHO5 *promoter and its associated transcriptionally active state can be inherited through DNA replication even in the absence of transcriptional activators. Transcriptional reinitiation from the nucleosome-depleted *PHO5 *promoter in the apparent absence of activators *in vivo *does not require Mediator. Notably, the epigenetic inheritance of the nucleosome-depleted *PHO5 *promoter through DNA replication does not require ongoing transcription.

**Conclusion:**

Our results suggest that there may be a memory or an epigenetic mark on the nucleosome-depleted *PHO5 *promoter that is independent of the transcription apparatus and maintains the promoter in a nucleosome-depleted state through DNA replication.

## Background

Histone removal, also termed nucleosome disassembly, from promoter regions is a recently-discovered mechanism of transcriptional regulation that is largely conserved throughout the eukaryotes [1-11]. The function of chromatin disassembly at promoters has been revealed from studies at the yeast *PHO5 *promoter. Histone removal is required to allow access of the general transcription machinery to the promoter in order to initiate transcription [1,12]. Conversely, repression of the *PHO5 *promoter is accompanied by chromatin reassembly [13]. Chromatin reassembly at the promoter is mediated by the histone chaperone Spt6 and is essential for transcriptional repression because the histones effectively compete with the general transcription machinery for occupancy at the *PHO5 *promoter [13].

**Figure 1:**
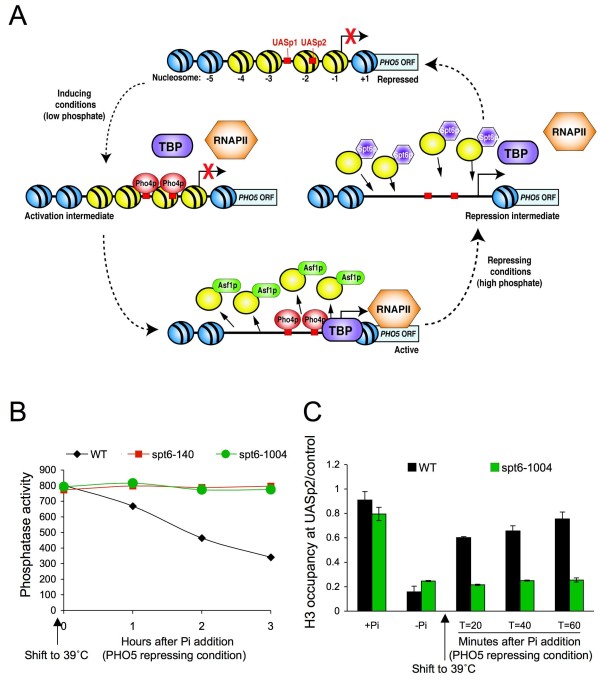
**Spt6-mediated chromatin assembly and transcriptional repression at the *PHO5 *promoter**. **(a) **UASp1 and UASp2 are binding sites for the Pho2 and Pho4 transactivators. During activation, chromatin disassembly of the four yellow nucleosomes is promoted by Asf1 to allow access of the general transcription machinery to the promoter. During repression, chromatin is reassembled over the *PHO5 *promoter by the histone H3/H4 chaperone Spt6 to compete with the general transcription machinery for DNA binding. **(b) **Strain JKT0010 (WT), JMY0002 (*spt6-140*), and MAY0067 (*spt6-1004*) were grown in phosphate-depleted media to activate *PHO5 *transcription. Following a 4 hour shift to 39°C to inactivate Spt6, phosphate was added as a signal for *PHO5 *repression. Samples were assayed for phosphatase activity at the indicated times after addition of phosphate. **(c) **Strain JKT0010 (WT) and MAY0067 (*spt6-1004*) were initially grown in phosphate-rich media (+Pi) where *PHO5 *is repressed, then shifted to phosphate-depleted media to activate *PHO5 *transcription (-Pi). Following a 2 hour shift to 39°C, phosphate was added. Samples were taken at the indicated times, and analyzed for histone occupancy at *PHO5 *UASp2 by chromatin immunoprecipitation (ChIP) analysis. The amount of immunoprecipitated DNA was determined by quantitative PCR. As a control, primer sets were used for TELVIR. Quantitation of H3 levels over the UASp2 region is a ratio of immunoprecipitated UASp2 product relative to the immunoprecipitated TELVIR product divided by the ratio of input UASp2 product relative to the ratio of input TELVIR product. Averages of three independent experiments are shown; error bars indicate the 95% confidence interval.

The yeast *PHO5 *gene encodes an acid phosphatase and its expression is tightly regulated by intracellular phosphate levels. In low phosphate conditions, the sequence-specific transactivator Pho4 is dephosphorylated causing it to localize to the nucleus where it binds the *PHO5 *promoter [14,15]. Pho4 binding to the DNA initiates depletion of the four positioned nucleosomes that normally reside over the *PHO5 *promoter including the Pho4 binding site termed UASp2 and the TATA box [16]. In repressing conditions (high phosphate), Pho4 is phosphorylated by Pho80-Pho85, which causes its export to the cytoplasm [14,17]. Loss of Pho4 from the promoter leads to the histones being reassembled by Spt6 at the *PHO5 *promoter [16] (Figure 1). We have previously shown that inactivation of Spt6 prior to addition of the signal for repression (phosphate) results in the *PHO5 *promoter remaining nucleosome-depleted and transcriptionally active *in vivo*, even though the activators no longer occupy the promoter [13]. This indicates that the main role of some transcriptional activators is to maintain promoters in a nucleosome-depleted state, which in turn indirectly allows the binding of the general transcription machinery to the core promoter.

In this work, we set out to determine whether the inducibly nucleosome-depleted *PHO5 *promoter and its associated transcriptionally active state can be inherited through DNA replication in the absence of the activators that originally initiated this state. We discovered that inactivation of the chromatin assembly factor Spt6 enables the *PHO5 *promoter to be maintained in a nucleosome-depleted state through DNA replication even when the activator that originally signaled for promoter chromatin disassembly was exported to the cytoplasm prior to DNA replication. Basically, we have achieved epigenetic inheritance of a nucleosome-depleted DNA state facilitating persistent transcription in the absence of transcriptional activators *in vivo*. Furthermore, we show that ongoing transcription is not required for the inheritance of this inducibly nucleosome-depleted promoter region. Our results suggest that there may be an epigenetic signal that can maintain promoters in a nucleosome-depleted state through replication.

## Results

In low phosphate conditions, binding of the transcriptional activator Pho4 to the *PHO5 *promoter is the signal for chromatin disassembly from the promoter. In high phosphate conditions, disengagement of Pho4 is the signal for Spt6-mediated chromatin reassembly of the *PHO5 *promoter (Figure 1(a)). Consequently, inactivation of the histone chaperone Spt6 using isogenic strains carrying either the *spt6-140 *or *spt6-1004 *temperature-sensitive allele prevented transcriptional repression of *PHO5 *in asynchronous cultures in response to the addition of phosphate (Figure 1(b)), even though we have previously shown that phosphate addition results in the rapid loss of Pho2 and Pho4 from the *PHO5 *promoter when Spt6 is inactivated [13]. This persistent *PHO5 *transcription under repressing conditions in the apparent absence of Spt6 and activators is due to failure to reassemble chromatin on to the *PHO5 *promoter (Figure 1(c)) [13], which enables continued access of the general transcription machinery to the promoter.

**Figure 2:**
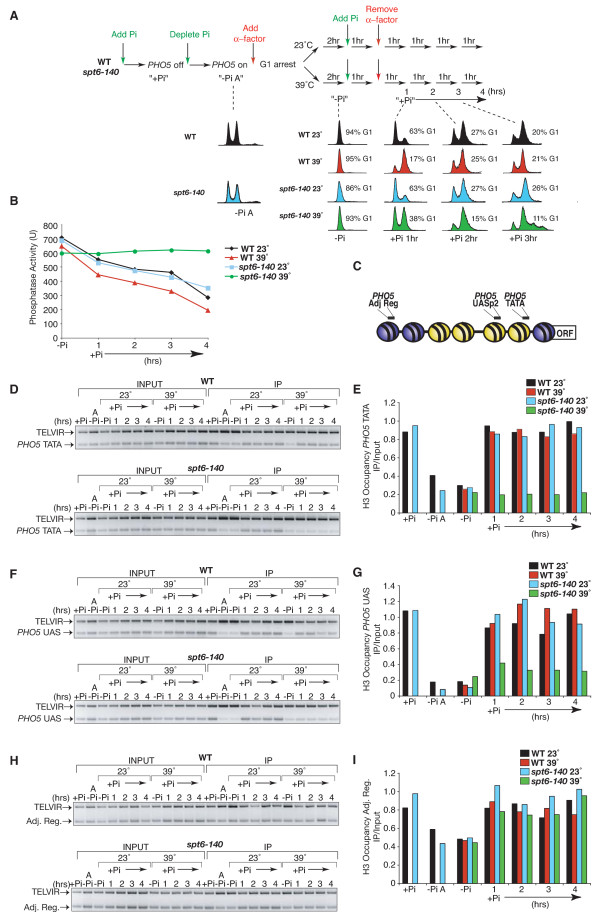
**The naked *PHO5 *promoter can be inherited through DNA replication even in the absence of the activators**. **(a) **Schematic of experimental outline, showing flow cytometry DNA content profiles for the strains JKT0010 (WT) and JMY0002 (*spt6-140*) at the indicated temperatures. '-Pi A' refers to asynchronously growing in phosphate-free media, while the remainder of the -Pi samples are at various stages of the cell cycle, as indicated by the flow cytometry analyses below. **(b) **Inactivation of Spt6 prevents repression of *PHO5 *even following DNA replication. Acid phosphatase activity of the *PHO5 *gene product was measured at the indicated times in the schematic shown in (a). **(c) **Schematic of locations of primer pairs used for the chromatin immunoprecipitation (ChIP) analyses shown below. **(d) **ChIP analysis of H3 occupancy at the *PHO5 *TATA region. **(e) **Quantitation of ChIP analysis of H3 occupancy at the *PHO5 *TATA region, normalized to the TELVIR internal control. **(f) **ChIP analysis of H3 occupancy at the *PHO5 *UAS region. **(g) **Quantitation of ChIP analysis of H3 occupancy at the *PHO5 *UAS region, normalized to the TELVIR internal control. **(h) **ChIP analysis of H3 occupancy at the *PHO5 *adjacent region. **(i) **Quantitation of ChIP analysis of H3 occupancy at the *PHO5 *adjacent region, normalized to the TELVIR internal control.

It is generally believed that all newly replicated DNA is reassembled into chromatin following DNA replication. Therefore, we asked whether the nucleosome-depleted *PHO5 *promoter in the absence of activators (that is, in the *spt6 *mutant +Pi condition) would also be reassembled into chromatin following DNA replication. To ensure passage through DNA replication had occurred, we arrested our wild type (WT) and *spt6-140 PHO5 *expressing cultures (-Pi) growing at 23°C in G_1 _phase with the mating pheromone alpha factor. Following cell cycle arrest, we split the cultures into two and placed one culture at 23°C and the other at 39°C to inactivate Spt6 (Figure 2(a)). After inactivation of Spt6, we added phosphate as the signal for Pho4 eviction from the promoter and removed the alpha factor to allow re-entry into the cell cycle. Passage through S-phase was confirmed by flow cytometry analysis (Figure 2(a)). The phosphatase activity of Pho5 confirmed that the WT strain at both temperatures and the *spt6 *strain at 23°C repressed *PHO5 *transcription upon addition of phosphate (Figure 2(b)). By contrast, the *spt6 *strain at 39°C failed to repress *PHO5 *transcription following passage through S-phase (Figure 2(b)), as in asynchronous cultures (Figure 1(b)), even though the activators should have disengaged from the promoter in response to phosphate addition [13]. As Spt6-mediated chromatin reassembly is absolutely required for *PHO5 *repression [13], the failure to repress *PHO5 *following DNA replication in the absence of functional Spt6 (Figure 2(b)) indicated that the chromatin may not be reassembled over the *PHO5 *promoter following DNA replication in *spt6 *mutants. We confirmed this prediction by chromatin immunoprecipitation (ChIP) analysis against the C-terminus of histone H3 using primer pairs spanning the TATA box, the UASp2 Pho4 binding site and the region adjacent to the *PHO5 *promoter (Figure 2(c)). This analysis showed that the chromatin fails to be reassembled on to the *PHO5 *promoter following DNA replication in the absence of Spt6, even though the activators (the signal to maintain chromatin disassembly) are no longer bound to the promoter (Figure 2(d)-2(g)) (13). Importantly, our results indicate that Spt6 does not mediate global replication-dependent chromatin assembly because the region adjacent to the nucleosome-depleted *PHO5 *promoter was reassembled into chromatin following DNA replication even when Spt6 was inactivated (Figure 2(h) and 2(i)). These results suggest that there is some factor or epigenetic mark at the *PHO5 *promoter that is signaling for it to remain nucleosome-depleted through DNA replication even in the absence of the transcriptional activators that initially triggered the nucleosome depletion.

**Figure 3:**
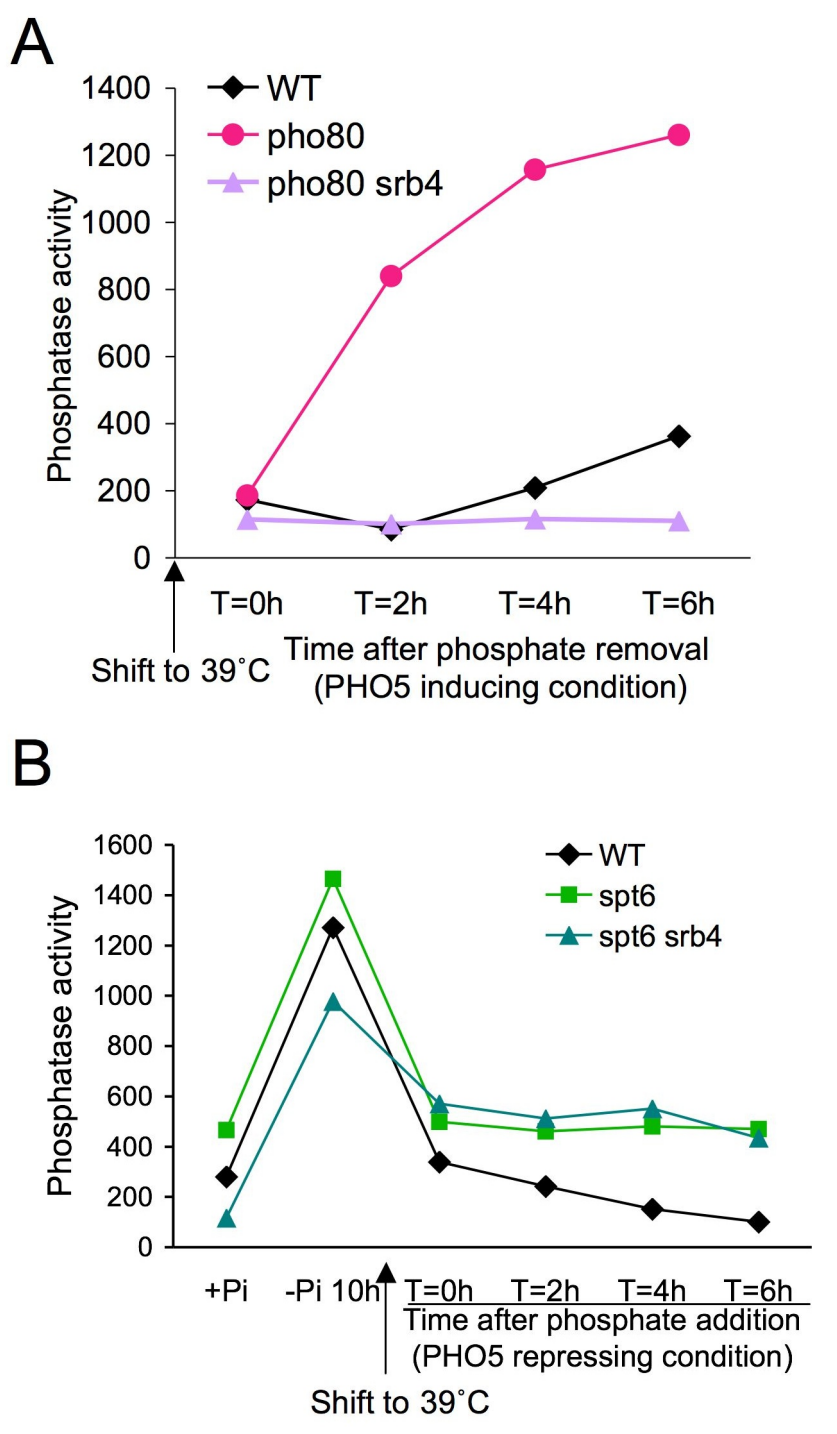
**Mediator is not required for *PHO5 *transcription in the absence of activators *in vivo***. **(a) **Mediator is required for transcriptional initiation of *PHO5 in vivo*. Strains JKT0010 (WT), ROY010 (pho80), and ROY011 (pho80 srb4) growing in phosphate containing media (*PHO5 *repressed) were shifted to 39°C in low phosphate media, followed by analysis of *PHO5 *induction via the phosphatase activity assay. **(b) **Mediator is not required for continued transcription from the nucleosome-depleted promoter in the absence of activators. Strains JKT0010 (WT), JMY002 (*spt6*), and SKW067 (*spt6 srb4*) were shifted to 39°C while *PHO5 *transcription was occurring in low phosphate media, followed by the addition of phosphate as the signal to repress *PHO5 *transcription and analysis of *PHO5 *induction via the phosphatase activity assay.

Next, we set out to identify the nature of the epigenetic mark or factor on the *PHO5 *promoter that signals for it to remain nucleosome-depleted even after DNA replication in the apparent absence of activators. Given that the histones are mostly depleted from the promoter, we focused on the transcription machinery rather than chromatin modifications or chromatin remodelers *per se*. First, we asked whether Mediator is required for continued transcription from the nucleosome-depleted promoter in the absence of activators, because Mediator is known to be required for transcriptional reinitiation *in vitro *[18,19]. To inactivate Mediator, we used a conditional mutant of the Mediator subunit Srb4. We first confirmed that Mediator is required for transcriptional initiation from the *PHO5 *promoter *in vivo*. As inactivation of Srb4 by a shift to 39°C blocks cell growth, we performed these experiments in the context of a temperature-sensitive mutation of *PHO80*, which allowed us to bypass the need for cell growth (in order to use up cellular phosphate stores) that is normally required for *PHO5 *induction. In this experiment, we clearly see that *PHO5 *is induced upon inactivation of Pho80 as expected, and that this induction of *PHO5 *requires Srb4 because it does not occur in the *pho80 srb4 *double mutant (Figure 3(a)). As such, Mediator is required for initiation of transcription from the *PHO5 *gene.

Next, we asked whether Mediator is required for the persistent transcription that occurs from the nucleosome-depleted *PHO5 *promoter in the absence of activators. Given that the nucleosome-depleted promoter can be maintained through DNA replication (Figure 2), all subsequent repression experiments were performed in asynchronously cycling cells. We shifted the *PHO5 *expressing cultures to 39°C to inactivate both Mediator and Spt6, followed by addition of the repressive signal (phosphate). Notably, we observed a repression of transcription that was due to the heat shock alone, as it was seen even before we added the phosphate to repress *PHO5 *transcription (Figure 3(b), at *T *= 0 hour). The reason why the heat shock alone partially represses *PHO5 *transcription is unclear, but it is accompanied by partial reassembly of the chromatin in an Spt6-independent manner upon the heat shock (see later). Notwithstanding, the completion of promoter chromatin assembly and the full transcriptional repression that is mediated by Spt6 were still apparent after adding phosphate to the WT and *spt6 *strain (Figure 3(b)). Interestingly, we reproducibly find that inactivation of Srb4 does not prevent the persistent transcription that occurs in the absence of Spt6 (Figure 3(b)). This result indicates that Mediator is not required for transcriptional reinitiation of activator-independent transcription from a nucleosome-depleted promoter *in vivo*. This is in contrast to the requirement for Mediator in transcriptional reinitiation of activator-independent transcription from naked DNA templates *in vitro *[18,19], suggesting that the *in vitro *transcription systems may not fully recapitulate the situation *in vivo*.

**Figure 4:**
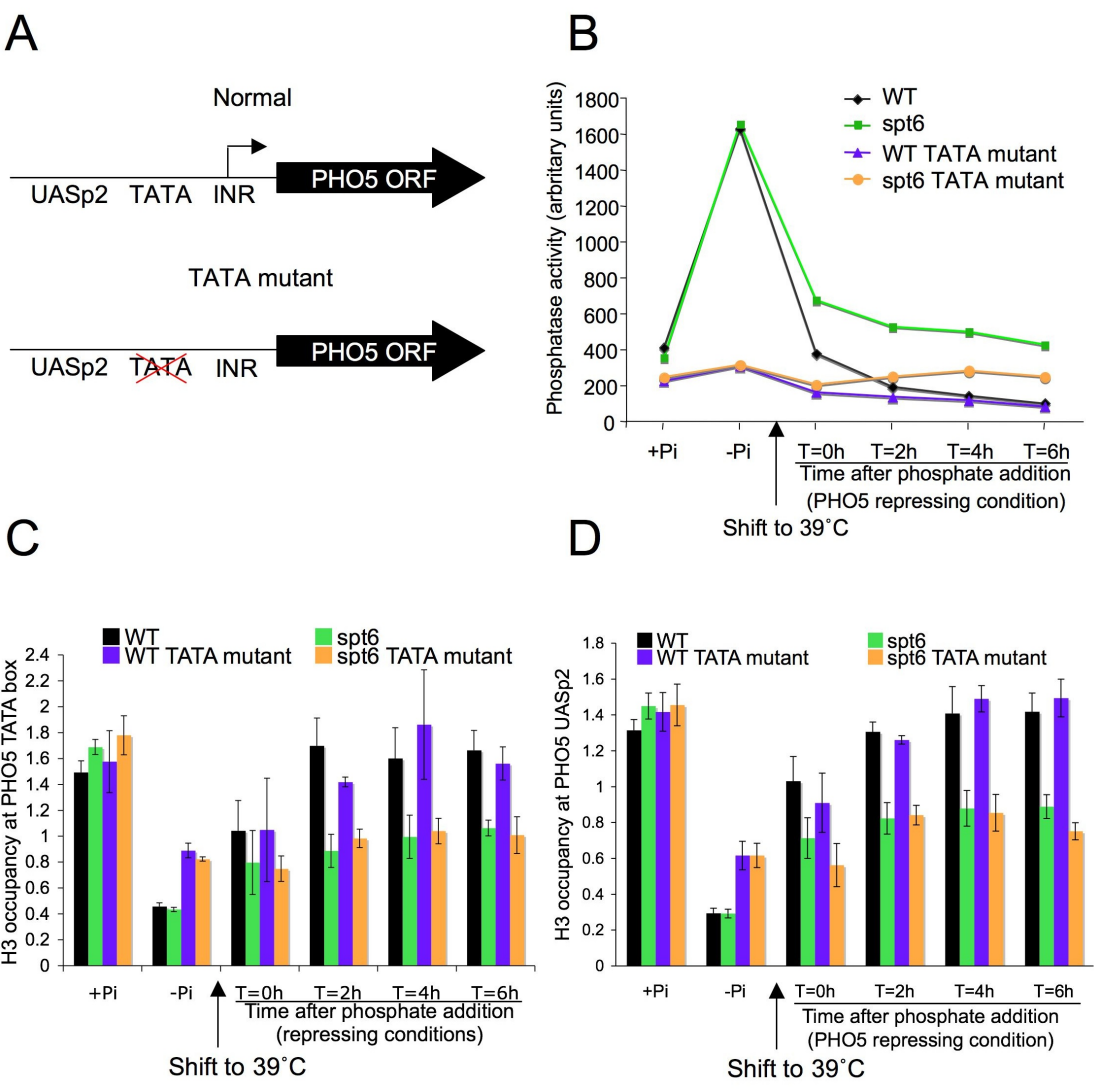
**Maintenance of the nucleosome-depleted *PHO5 *promoter does not require persistent transcription**. **(a) **Schematic of constructs used for removal of the analyses below. **(b) **JKT0010 (WT), ROY008 (*spt6*), JQS002 (WT TATA mutant), and ROY009 (spt6 TATA mutant) previously growing in phosphate containing media (-Pi) were subject to growth in the absence of phosphate (-Pi) to stimulate removal of nucleosomes from the *PHO5 *promoter, followed by a temperature shift to 39°C to inactivate Spt6, followed by addition of phosphate to stimulate removal of the activators from the promoter. Samples at the indicated times after phosphate addition, or in the indicated media were taken for phosphatase activity assays. **(c) **Samples of strains JKT0010 (WT), ROY008 (*spt6*), JQS002 (WT TATA mutant), and ROY009 (*spt6 *TATA mutant) taken from the same time course in (b) were assessed for histone occupancy over the TATA box of the *PHO5 *promoter. Data were normalized to the *GAL1 *promoter region as a control region, and to the input samples. The average and standard deviation of three independent experiments are plotted. **(d) **Samples of strains JKT0010 (WT), ROY008 (*spt6*), JQS002 (WT TATA mutant), and ROY009 (*spt6 *TATA mutant) taken from the same time course in (b) were assessed for histone occupancy at the UASp2 binding site of the *PHO5 *promoter. Data were normalized to the *GAL1 *promoter region as a control region, and to the input samples. The average and standard deviation of three independent experiments are plotted.

Next, we asked whether transcription itself was required for the maintenance of the nucleosome-depleted promoter through DNA replication in the absence of transcriptional activators *in vivo*. To prevent *PHO5 *transcription, we used a strain with a point mutation in the *PHO5 *TATA box [20] (Figure 4(a))). As expected, mutation of the *PHO5 *TATA box prevented transcription from *PHO5 *(Figure 4(b)). Next, we examined whether mutation of the *PHO5 *TATA box influenced the failure of chromatin to be reassembled onto the promoter following DNA replication in the *spt6 *mutant upon addition of phosphate (the signal for repression). We found that the promoters of the TATA box mutants were not disassembled quite as well as the normal TATA box strains (Figure 4(c) and 4(d)). Upon addition of phosphate as the signal for repression, the extent of chromatin reassembly in the *spt6 *mutants with and without the TATA box were equally impaired as compared with the efficient chromatin reassembly observed in the WT and the WT TATA mutant at both the TATA box and the UASp2 site (Figure 4(c) and 4(d)). These results indicate that transcription from the *PHO5 *promoter is not required to maintain the promoter in a nucleosome-depleted state through DNA replication.

## Discussion

### Epigenetic inheritance of an inducibly nucleosome-depleted promoter in the apparent absence of transcriptional activators

We have developed a system that enables epigenetic inheritance of expression of the *PHO5 *gene in the apparent absence of transcriptional activators by maintaining the promoter in a nucleosome-depleted state. We achieved this situation by inactivating the Spt6 chromatin assembly factor prior to addition of the repression signal (phosphate) for *PHO5 *transcription. The phosphate signals for the activators to dissociate from the *PHO5 *promoter, while inactivation of Spt6 prevents reassembly of the *PHO5 *promoter into chromatin. Surprisingly, we found that this naked *PHO5 *promoter remains naked through DNA replication, even though the original signal for chromatin disassembly, the activators, left the promoter prior to DNA replication. This epigenetic inheritance of the nucleosome-depleted promoter does not require the Pho2 and Pho4 activators, Mediator, nor transcription *per se*, suggesting that an epigenetic mark is retained on the promoter to maintain the nucleosome-depleted state through DNA replication.

*PHO5 *transcription normally requires high levels of unphosphorylated Pho4 and Pho2 to be bound to the *PHO5 *promoter. The fact that *PHO5 *repression occurs when the Pho4 and Pho2 activators leave the promoter indicates that they do not function via a 'hit-and-run' mechanism. Furthermore, all the evidence indicates that Pho4 and Pho2 no longer occupy the *PHO5 *promoter during the epigenetic inheritance of the nucleosome-depleted promoter in the *spt6 *mutant. Upon phosphate addition, the Pho80-Pho85 cyclin-cyclin-dependent kinase complex phosphorylates Pho4, resulting in seemingly complete nuclear export of Pho4 by 3 to 6 minutes after phosphate addition [21]. Even when phosphorylated Pho4 is made to remain in the nucleus it does not activate *PHO5 *transcription [22]. This is because phosphorylated Pho4 fails to bind to Pho2, and the interaction between Pho4 and Pho2 is required for recruitment of Pho4 to the *PHO5 *promoter [23]. Our previous studies in the same *spt6 *mutant strain and same growth conditions that we used in this work demonstrated that 1 hour after adding phosphate to the media Pho2 and Pho4 no longer occupied the *PHO5 *promoter (by ChIP analysis and *in vivo *dimethyl sulfate footprinting analyses) and Pho4 had left the nucleus (by Pho4 green fluorescent protein localization analysis) [13]. It should be noted that even without synchronization of the cells prior to DNA replication with alpha factor, the extended period of growth in phosphate-depleted media that is required to activate *PHO5 *transcription leads to cell synchronization prior to DNA replication. This is because after the cells have used up their polyphosphate stores they are unable to obtain any more phosphate to make nucleotides, resulting in arrest prior to DNA replication. Conversely, replication resumes upon addition of phosphate, which is the stimulus for *PHO5 *repression, because nucleotide synthesis resumes. As such, the continued *PHO5 *transcription that we observed previously in the absence of activators in the *spt6 *mutant without alpha factor arrest of the cells reflected the maintenance of the nucleosome-depleted and transcriptionally active state of the *PHO5 *promoter through DNA replication [13].

It has always been assumed that the entire genome is rapidly reassembled into chromatin following every round of DNA replication. The majority of persistently nucleosome-free regions in the genome are thought to be nucleosome-free due to their AT-rich sequences, which are rigid and therefore incorporated poorly into nucleosomes [24-27]. By contrast, nucleosomes are depleted from promoters in response to transcriptional activator binding. Following DNA replication, transcriptional activators presumably rebind to their sites and signal for the disassembly of chromatin from promoters again. However, in our system the *PHO5 *promoter remains nucleosome-depleted through DNA replication even in the absence of activators (Figure 2). As our system requires that Spt6 be inactivated, it was essential to show that Spt6 is not mediating global replication-dependent chromatin assembly, which we did by demonstrating that the region adjacent to the *PHO5 *promoter reassembles into chromatin following DNA replication in the absence of functional Spt6 (Figure 2(h) and 2(i)). To our knowledge, this is the first example of a system for studying the epigenetic inheritance of an inducibly nucleosome-depleted DNA state. Mechanistically, we do not know if the histones are reassembled on to the *PHO5 *promoter by the global replication-dependent chromatin reassembly apparatus and then rapidly disassembled after replication, or whether the *PHO5 *promoter was never reassembled into chromatin. Distinction between these two possibilities will be important for understanding the molecular basis of the epigenetic inheritance of a nucleosome-depleted state.

The replication machinery is a highly processive complex that rapidly copies huge numbers of bases while competing with histones, DNA-binding proteins, and transcription factors. Although the replication machinery generally wins out, there are examples of site-specific barrier elements in eukaryotes that mediate replication termination to prevent collisions between the replication machinery and transcription machinery [28,29]. Replication forks also pause at stable protein-DNA complexes and within the open reading frames of highly transcribed genes in yeast [30]. However, the *PHO5 *promoter, along with the rest of the genome, does get replicated and it is hard to imagine how any factor could remain bound to the DNA while the DNA is being threaded through the replication machinery [31]. Indeed the Rrm3 DNA helicase moves with the yeast replication machinery seemingly to remove stably bound proteins from the DNA [32]. Although it has not yet been conclusively addressed for eukaryotic RNA polymerase II transcription, the general transcription machinery is also presumably displaced by passage of the DNA replication machinery. Therefore, following DNA replication, the general transcription machinery reassociates on to the promoter for transcription to proceed and is normally facilitated by transcriptional activators. In our system, the general transcription machinery reassembles on to the nucleosome-depleted *PHO5 *promoter in the absence of transcriptional activators *in vivo *to give efficient transcriptional initiation after DNA replication. As such, activators are not required for transcriptional initiation *in vivo *if a promoter is nucleosome-depleted, extending our previous observation that activators are not required for transcriptional reinitiation *in vivo *if a promoter is nucleosome-depleted [13]. Similarly, our work demonstrates that Mediator is not required for transcriptional reinitiation in the absence of activators *in vivo *at the nucleosome-depleted *PHO5 *promoter. This suggests that a critical role of Mediator *in vivo *is to help activators open up the chromatin structure in order to enable the general transcription machinery to gain access to the core promoter. This idea is consistent with the recently reported role of Mediator in histone H3 phosphorylation and acetylation [33], revealing a mechanism whereby Mediator helps open up the chromatin structure.

### What is the epigenetic mark that specifies a promoter to remain nucleosome-depleted through DNA replication?

We have ruled out the possibility that transcription *per se *specifies that the *PHO5 *promoter should remain nucleosome-depleted, because mutation of the *PHO5 *TATA box does not prevent inheritance of the nucleosome-depleted promoter through DNA replication. Notably, the TATA box is not required for chromatin disassembly from the *PHO5 *promoter [34] (Figure 4(c) and 4(d)). It is also possible that a component of the general transcription machinery is bound to the mutant TATA box promoter that prevents chromatin reassembly following DNA replication. Even so, it would be unlikely that such a factor could block chromatin reassembly over both the TATA box region and the adjacent nucleosome to equivalent degrees (as in Figure 4(c) and 4(d)). It is noteworthy that virtually no RNA polymerase II is detected on the mutant TATA box *PHO5 *promoter [20]. Some activators have been shown to function by a hit-and-run mechanism. For example, the Swi5p activator occupies the HO promoter for only 5 minutes, where it mediates recruitment of SAGA and SWI/SNF, which then allows subsequent recruitment of the activator SBF [35]. However, it is unlikely that Pho4 is acting in this hit-and-run mechanism, as Pho4 is required to occupy continuously the *PHO5 *promoter to maintain *PHO5 *transcription, and its dissociation is normally the signal for repression (unless promoter chromatin reassembly is prevented by inactivation of Spt6).

It is also possible that the epigenetic mark for inheritance of nucleosome-depleted DNA is the absence of nucleosomes *per se*. If chromatin reassembly was directed by the inherited old nucleosomes that are transferred locally to the newly replicated DNA, then the failure to inherit old nucleosomes may be sufficient to maintain the promoter in a nucleosome-depleted state through replication. However, opposing the idea that lack of histones on the parental DNA dictates lack of histones on the newly replicated DNA is the fact that replication-coupled chromatin assembly can be achieved on naked DNA templates *in vitro *[36,37].

Histone modifications are another way to potentially propagate epigenetic information through DNA replication because the pattern of histone modifications has recently been shown to be preserved through DNA replication [38], when old nucleosomes are distributed randomly on both sides of the fork, with the newly synthesized histones interspersed. In this model, the bromodomains, chromodomains, and so on [39] of chromatin-modifying enzymes would recognize their cognate modifications on the segregated parental histones, permitting the propagation of specific 'histone codes' to adjacent newly assembled nucleosomes following DNA replication [40]. It is possible that histone modifications are involved in the mechanism of epigenetic inheritance of nucleosome-depleted promoters, because the *PHO5 *promoter is not completely disassembled of chromatin upon transcriptional activation. On average, only three of the four nucleosomes between positions -1 and -4 of the *PHO5 *promoter are removed from the active promoter [41,42]. Therefore, it is possible that the nature of the epigenetic mark that specifies a nucleosome-depleted *PHO5 *promoter may be a specifically modified histone that remains in the -1 to -4 nucleosome positions. Alternatively, the nucleosomes flanking the disassembled region may be specifically modified and signal that the intervening region should remain disassembled. Alternatively, a non-histone factor bound to the *PHO5 *promoter could signal for chromatin disassembly.

Nucleosomes with dimethylated K36 H3 are refractory to nucleosome disassembly [43], making its depletion an attractive mark for nucleosome disassembly. Consistent with this idea, we find that induction of *PHO5 *is extremely rapid in yeast deleted for the gene encoding the Set2 methylase for H3 K36 (data not shown). Interestingly, *spt6-1004 *mutants, but not *spt6-140 *mutants, lack all H3 K36 methylation presumably due to instability of the Set2 protein [44,45]. It will be interesting to investigate in the future whether the histone-depleted *PHO5 *promoter is additionally depleted of H3 K36 me2. H3 K36 is a mark for subsequent histone deacetylation [46,47], so its absence would lead to a more acetylated and potentially more readily disassembled promoter. It is also quite likely that a single histone modification will not be sufficient to trigger chromatin disassembly, as these modifications are fairly widespread on the genome. Future studies should reveal the molecular basis for the epigenetic inheritance of the nucleosome-depleted *PHO5 *promoter, which may serve as a model for understanding the epigenetic inheritance of transcriptional programs in higher eukaryotes that are established by hit-and-run transcriptional activators.

## Conclusion

We have found that the inducibly-nucleosome depleted yeast *PHO5 *promoter is not reassembled into chromatin following DNA replication in the absence of the activators that originally signaled for the nucleosome-depletion. As such, this unique system of epigenetic inheritance should facilitate the discovery of the epigenetic mark that maintains the nucleosome-depleted promoter and its transcriptional activity in the absence of activators.

## Methods

### Yeast strains and media

All media used were either YPD (high phosphate) or phosphate-depleted YPD media, made as previously described [48]. Temperature shifts were achieved by spinning down cells and adding prewarmed media to the cell pellet, followed by 4 hours incubation at 39°C prior to addition or removal of phosphate. The doubling times for the WT strain at 23°C is 120 minutes and for the *spt6-140 *strain at 23°C is 180 minutes. The doubling time for the WT strain at 39°C is 190 minutes and for the *spt6-140 *strain at 39°C is 320 minutes. JKT0010, MAT = a his3-11 leu2-3, 112 lys2 trp1-1 ura3-1 bar1::LEU2 w303, is isogenic to ROY008 MAT = a his3-11 leu2-3, 112 lys2 trp1-1 ura3-1 bar1::LEU2 spt6-140 w303. JMY002, which carries the spt6-140 ts mutation has been described previously [13]. MAY0067 carries the spt6-1004 allele and is MAT = a his3-11 leu2-3, 112 lys2 trp1-1 ura3-1 bar1::LEU2 spt6-1004 w303. ROY0010 is MAT = a ade2-1 trp1-1 can1-100 leu2-3, 112 his3-11, 15 ura3 GAL+ pho81::TRP1B can1::pPHO5-CAN1 srb4::KANMX6 pRY2844 (LEU2 SRB4+). ROY0011 is MAT = a ade2-1 trp1-1 can1-100 leu2-3, 112 his3-11, 15 ura3 GAL+ pho81::TRP1B can1::pPHO5-CAN1 srb4::KANMX6 pRY2882 (LEU2 srb4-138). The pho80 srb4 double ts mutant was made by transformation of plasmids pRY2844 and pRY2882 and deletion of the endogenous SRB4 locus by deletion cassette replacement, as described previously [49]. Strain SKW066 is derived from strain JMY002, but additionally has the *BAR1 *gene deleted by insertion of the KanMX6 marker. Strain Z628 carrying the srb4 ts mutant was described previously [49]. Strain KLY042 is derived from strain Z628, but additionally carries SWI1-9myc::URA3 and was described previously [50]. JQS002 carries the *PHO5 *TATA box mutation and was described previously [20]. ROY009 was derived from JQS002 by insertion of the spt6-140 allele in place of the endogenous *SPT6 *gene by two-step integration. The *spt6 srb4 *double ts mutant was generated by dissection of tetrads from diploids that were heterozygous for the two temperature-sensitive mutations. Unless described otherwise, strains were constructed using standard single-step integration methods.

### Acid phosphatase activity assays

Approximately 5 ml of cells were collected by centrifugation and washed with cold 0.1 M sodium acetate pH 3.6, then resuspended in 500 μl of the same buffer. To determine the number of cells used for each reaction, 100 μl of the cells were diluted 1:10 in ddH_2_O and read at optical density (OD) 600 nm. For each sample reaction, another 100 μl of washed cells were diluted 1:5 for a total volume of 500 μl in the same sodium acetate buffer and prewarmed for 10 minutes at 30°C. A 500 μl sample of buffer alone was also included as a control, as well as an appropriate volume (500 μl per reaction) of freshly made substrate (nitro phenyl phosphate 0.0742 g/10 ml 0.1 M sodium acetate pH 3.6). After warming, 500 μl of substrate was added to each reaction sample and incubated at 30°C for 10 minutes, at which time 250 μl of stop solution, 1 M Na_2_CO_3_, was added. Samples were centrifuged for 1 minute and then read at OD 410 nm. Phosphatase activity was calculated as [(OD 410 × 1,000)/(OD 600 × volume cell lysate used (μl) × incubation time (minutes)]. Although single phosphatase time courses are shown in the figures, the results and general trends were all reproduced independently multiple times.

### ChIP analysis

Yeast cultures (150 ml) were grown to a density of 1 × 10^7 ^cells/ml and treated with 1% formaldehyde (final concentration) for 20 minutes at room temperature. Cross-linking was quenched by addition of glycine to a final concentration of 125 mM. Cells were sedimented and washed twice in ice-cold Tris-buffered saline (150 mM NaCl, 20 mM Tris HCl pH 7.5). Cells were resuspended in 400 μl lysis buffer (0.1% sodium deoxycholate, 1 mM ethylenediaminetetraacetic acid, 50 mM 4-(2-hydroxyethyl)-1-piperazineethanesulfonic acid pH 7.5, 140 mM NaCl, 1% Triton X-100), an equal volume of 0.5 mm glass beads were added, and the cells were vortexed for 10 minutes at 4°C. Chromatin was sheared with a Branson Sonifier 450 to an average size of 500 base pairs. Immunoprecipitations were performed using 2.5 μl of the C-terminus anti-histone H3 (Abcam #ab1791) overnight at 4°C as described previously [1]. For Figures 1 and 2, the linear range of template for multiplex polymerase chain reaction (PCR) was determined empirically and PCR-amplified products were quantitatively measured using Labworks (UVP Inc., Upland, CA, USA) as described previously [1].

The ChIP quantitation in Figure 4 was performed by real-time PCR using a Roche Applied Sciences Light Cycler 480. The linear range of PCR templates was determined by performing a 10-fold serial dilution standard curve, which usually proved a 1:10 dilution was sufficient. Each sample was analyzed in triplicate using 10 μl reactions in a 384-well plate format. The thermal profile was as follows: (1) denaturation at 95°C for 10 minutes; (2) run cycle of 95°C for 15 seconds then 60°C for 1 minute for 50 to 60 cycles; then (3) cooling at 40°C for 30 seconds. Each ChIP sample was normalized to its respective Input samples (to account for the number of cells taken), as well as a control region called GAL1/10 whose histone occupancy is regulated by glucose, not phosphate levels.

Primers and Taqman probes used were:

PHO5 UASp2 A: GAATAGGCAATCTCTAAATGAATCGA

PHO5 UASp2 B: GAAAACAGGGACCAGAATCATAAATT

PHO5 UASp2 probe: FAM-ACCTTGGCACTCACACGTGGGACTAGC-MGB

GAL1/10 A: GACGCACGGAGGAGAGTCTT

GAL1/10 B: CGCTTAACTGCTCATTGCTATATTG

GAL1/10 probe: FAM-CGCTCGGCGGCTTCTAATCCG-MGB.

## Abbreviations

ChIP: chromatin immunoprecipitation; OD: optical density; PCR: polymerase chain reaction; WT: wild type

## Competing interests

The authors declare that they have no competing interests.

## Authors' contributions

RO and MA performed the experiments. JKT participated in the overall design and coordination of the study and wrote the manuscript. All authors read and approved the final manuscript.
